# Adaptation of an Evidence-Based HIV Prevention Intervention to Address the Needs of Transgender Latina Women

**DOI:** 10.1089/trgh.2024.0054

**Published:** 2024-09-12

**Authors:** Jane J. Lee, Yesenia Cruz, Joel Aguirre, Martha Zuniga, Juliann Li Verdugo, Pedro Tomas-Domingo, Tracey Hernandez, E. Roberto Orellana, Lisa M. Kuhns, Robert Garofalo, Susan M. Graham

**Affiliations:** 1 School of Social Work, University of Washington, Seattle, Washington, USA.; 2 Entre Hermanos, Seattle, Washington, USA.; 3 School of Public Health, University of Washington, Seattle, Washington, USA.; 4 Division of Adolescent and Young Adult Medicine, Ann & Robert H. Lurie Children’s Hospital of Chicago, Chicago, Illinois, USA.; 5 Department of Pediatrics, Northwestern University Feinberg School of Medicine, Chicago, Illinois, USA.; 6 Department of Medicine and Global Health, University of Washington, Seattle, Washington, USA.

**Keywords:** adaptation, HIV prevention, intervention development, transgender Latina women

## Abstract

Transgender Latina women are disproportionately affected by human immunodeficiency virus (HIV) in the United States. Given unique needs, elevated risk, and a paucity of tailored interventions, we adapted an evidence-based HIV prevention program for this population. Following a needs assessment with transgender Latina immigrants, we adapted *Project LifeSkills*, a six-session group intervention for transgender youth, into a modified program titled *Somos Chingonas*, using methods from the ADAPT-ITT framework. Our adaptation process underscores the critical role of community involvement in developing tailored interventions for minority communities. Future research will pilot and evaluate the modified intervention to examine its impact on HIV prevention outcomes.

## Introduction

Transgender women in the United States are disproportionately affected by human immunodeficiency virus (HIV), with recent trends demonstrating a 20% increase in new HIV diagnoses from 2014 to 2019 in this demographic.^1,2^ The rise in HIV infections in this group emphasizes the urgency to address HIV transmission among transgender women in the United States and globally and the importance of effective prevention interventions to reduce disparities.

Within this broader context, transgender Latina women in the United States experience an elevated burden of HIV.^2,3^ According to the U.S. National HIV Behavioral Surveillance report for 2019–2020, Latinas constitute over a third of all HIV diagnoses among transgender women, and more than one in three transgender Latina women report being HIV positive.^2^

This heightened vulnerability is driven by a complex interplay of systemic and intersectional factors, including structural barriers, due to identification as racial and ethnic minorities, gender minorities, and immigrants.^4^ For example, stigma and discrimination are pervasive barriers to HIV prevention efforts on the systemic level, and limited health care coverage and legal protections among these communities further elevate the risk for HIV.^4–6^ Recognizing and addressing these multifaceted challenges is crucial for advancing equitable health outcomes within the transgender Latina community.

Despite the clear challenges that transgender Latina women face in accessing health and HIV-related services, there is a paucity of tailored evidence-based research focusing on addressing their unique HIV prevention needs.^4–7^ Among the existing evidence-based interventions (EBIs) and best practices for HIV prevention compiled by the Centers for Disease Control and Prevention (CDC) through December 2023, no interventions to our knowledge focus exclusively on transgender Latina women.^8^ To address this gap and optimize outcomes for transgender Latina women, we sought to adapt an evidence-based HIV prevention intervention for this population.

## Methods

### Overview

This study used the first six steps of the ADAPT-ITT method,^9^ which include (1) Assessment, (2) Decision, (3) Administration, (4) Production, (5) Topical experts, and (6) Integration. The adaptation was conducted in partnership with Entre Hermanos, a community-based organization based in Seattle, Washington, that serves the Latino/a/e/x lesbian, gay, bisexual, transgender, and queer (LGBTQ+) community.

All study procedures were reviewed and approved by the Institutional Review Board at the University of Washington. Informed consent was obtained from all study participants, and all research was completed in accordance with the Declaration of Helsinki, as revised in 2013. The research team was comprised of community members and researchers with extensive training in qualitative research and an understanding of the transgender Latina community. Adaptation of the intervention took place between September 2022 and July 2023, spanning 11 months.

### ADAPT-ITT steps

#### Assessment

First, we conducted a needs assessment with the target population, involving in-depth interviews (*n* = 10) with transgender Latina women purposively recruited by Entre Hermanos. These interviews evaluated HIV-associated behavioral and psychosocial risks, as well as preferences for intervention content and delivery. The goal was to explore participant perspectives and comprehend how their various identities, social processes, and the sociopolitical climate influence their health needs and experiences with health care access. In-depth interviews were audio-recorded, transcribed, and analyzed using a thematic approach.^10^ Results have been published.^10^

#### Decision

Based on the insights gained from the needs assessment, we moved to the decision step, where we reviewed existing CDC-defined EBIs for HIV prevention. The selection of the intervention for adaptation was guided by the “goodness of fit” between the original EBI and the needs of transgender Latina women. All materials were translated into Spanish by Entre Hermanos with attention to cultural relevance and appropriateness.

#### Administration

The third step involved administering a theater test in Spanish with transgender Latina women (*n* = 7) to gather feedback on the content, material, and delivery of the early adapted version of the intervention. Theater test participants were purposively recruited by Entre Hermanos staff and excluded participants in Assessment step interviews. Participants were eligible if they were 18+ years old, identified as transgender women, and identified as Hispanic/Latina. The theater test took place at Entre Hermanos and was facilitated by two staff members in Spanish, given the language preferences of the community. The theater test included presenting intervention modules and activities, followed by a focus-group style discussion described below, to collect critiques and identify additional materials and activities to enhance relevance and efficacy.

#### Production

Following the theater test, we conducted a brief focus group-style discussion with theater test participants that lasted approximately one hour. The focus group discussion was facilitated by the two staff members who conducted the theater test and followed an unstructured guide that asked for feedback on the format and structure of the intervention, the intervention content, and the delivery of the intervention. This feedback informed the adaptation process, guiding the modification of materials and development of a comprehensive plan outlining the adaptation’s aim, core elements from the original EBI, and descriptions of new materials and characteristics of the adapted intervention.

#### Topical experts

The fifth step involved obtaining perspectives from topical experts (*n* = 5). This was achieved through a series of brief interviews and the presentation of adapted materials to researchers (*n* = 2) and community members at Entre Hermanos (*n* = 3) who are included as authors of this article. Interviews lasted ∼1 h and took place in-person or via Zoom. The interviews followed an unstructured guide to obtain perspectives on the appropriateness of the adapted intervention format, structure, content, and delivery. Feedback from topical experts was obtained through detailed note-taking during the interviews that documented reactions, suggested changes, and revisions.

#### Integration

The final step in the adaptation process centered on integrating content and feedback provided by topical experts into the adapted EBI. Feedback from the topical experts was compiled and summarized, then discussed at in-person meetings of the study team, which included researchers and community members at Entre Hermanos, to inform any additional changes. This iterative process aimed to refine the intervention and enhance its alignment with the specific needs and preferences of transgender Latina women in the context of HIV prevention. A shared, running document of intervention materials and edits was maintained throughout all adaptation steps.

## Results

### Intervention selection

Published findings from the Assessment interviews, available elsewhere,^10^ underscored the diverse forms of rejection and discrimination faced by transgender Latina individuals within specific contexts and environments. Interviewees highlighted multiple barriers to reducing their HIV risk and accessing HIV prevention services and a need for increased social support within the community and the adoption of status-neutral approaches to HIV service provision, to mitigate stigma associated with seeking care.^10,11^

Taking into account the needs and experiences of transgender Latina women and available EBIs, we opted to adapt the *Project LifeSkills* intervention.^12,13^ Developed by Dr. Robert Garofalo and colleagues,^13^ *Project LifeSkills* is a six-session group-level intervention originally designed for transgender youth. It aims to convey basic HIV risk and transmission information, foster motivation for self-protection, and enhance behavioral skills (e.g., condom use and assertive communication) through an empowerment-based approach.^12,13^

Our selection was based on several factors: (1) the group-level format, addressing the need for social connection in the transgender Latina community; (2) the intervention’s theoretical basis in Information–Motivation–Behavioral Skills^14^ and an empowerment-based approach, aligning with our goal to promote HIV-related prevention behaviors that empower the community; and (3) the EBI’s focus on young transgender women, complementing our aim to address challenges to sexual safety within the transgender Latina community, including among young adults as they increase sexual activity and related risk.

The decision to adapt *Project LifeSkills* was guided by its compatibility with our goals. By leveraging *Project LifeSkill*’s existing framework and resources, we could also streamline the adaptation process while maximizing the efficiency of resource allocation. This strategic alignment ensured that our intervention efforts would be effective as well as sustainable, enabling us to allocate resources to support the diverse needs of the transgender Latina community.

### Theater testing and focus group adaptations

Our theater test of the adapted EBI involved transgender Latina women (*n* = 7) who ranged in age from 25 to 62 years. Table 1 provides the demographic characteristics of the theater testing participants. This phase revealed the need for additional materials, content revisions, and integrated activities, informing modifications to intervention format, structure, content, and delivery (Table 2).

**Table 1.** Demographic Characteristics of Transgender Latina Theater Testing Participants (*n* = 7)

Characteristics	*N* (%)
Age (M)	45.2 years (range: 25–62)
Country of origin	
Mexico	5 (71.4)
Venezuela	1 (14.3)
El Salvador	1 (14.3)
Education	
Elementary school	2 (28.6)
Middle school	1 (14.3)
Some college/university	3 (42.8)
College degree	1 (14.3)
Annual income	
$0–19,999	4 (57.1)
$20,0000–39,000	1 (14.3)
$40,000+	2 (28.6)
Immigration status	
Temporary resident	2 (28.6)
Eligible immigrant	4 (57.1)
Legal permanent resident	1 (14.3)
Language	
Only Spanish	1 (14.3)
Spanish more than English	6 (85.7)
Health insurance	
Yes	4 (57.1)
No	3 (42.8)
HIV status	
Positive	3 (42.8)
Negative	4 (57.1)

HIV, human immunodeficiency virus; M, mean.

**Table 2.** Summary of Evidence-Based Intervention Adaptation and Changes Based on ADAPT-ITT Steps

Format and structure	
In-person and virtual interactions	Maintain six in-person sessions held in a community safe space and include creation of a WhatsApp group for participants to connect virtually throughout intervention participation.
Meals	Provide snacks and food for participants to facilitate participant interaction and support the overall community feel of the group. Ensure culturally appropriate foods are available.
Length of sessions	Offer 2-h sessions with additional time available for mingling and participant connection before and after sessions.
Schedule	Deliver sessions biweekly to provide adequate time to process materials and skills learned, and consider a start time that ensures adequate time for travel after work hours.
Intervention content	
Overall content	Maintain session topics including (1) stereotypes, self-esteem, and mental health; (2) harassment, violence, discrimination, and stigma; (3) safe housing, employment, education, and health care; 4) self-injections, hormone use, and HIV myths; (5) alcohol use, substance use, sex work, and unwanted sex; and (6) discussion, review, and celebration.
Additional content	Add content to address police/law enforcement interactions, gender dysphoria, and trauma-informed practices. Enrich content on HIV testing, PrEP, and ART (already included in initial intervention but enriched to encourage HIV service engagement).
Terms and language	Translate content into Spanish and ensure understanding of colloquial terms/slang used in different Spanish-speaking countries.
Delivery	
Activities	Maintain interactive activities (e.g., worksheets, roleplays, true or false games, flashcards, and group share-out discussions). Allow for sharing of experiences and opinions.
Intervention style	Maintain the dynamic and interactive style of program and include opportunities for participation, share-out discussions, and use of humor and colloquialisms to maintain a welcoming space.
Role plays	Develop and include scenarios and roleplays that reflect real-life experiences and situations of transgender Latina participants.
Facilitators	Employ a transgender Latina woman facilitator to allow for trust and mutual understanding as well as a shared understanding of experiences.
Participants	Allow transgender Latina women from any Spanish-speaking Latin American countries and at any stage of their transition to participate in the sessions to be able to make the program more inclusive and allow for more cultural adaptation. Consider inclusion of participants’ partners (or perspectives of partners) into a conversation as part of a session.

ART, antiretroviral therapy; PrEP, pre-exposure prophylaxis.

While retaining the six-session, group-based format and theoretical framework, we adapted session-specific content for increased relevance and cultural sensitivity based on focus group feedback. Participants shared positive feedback on intervention topics and materials and suggested that we include images and content that reflected their identities as transgender Latinas. For example, content on housing and employment was tailored to address the specific challenges faced by this community, such as language barriers, lack of documented immigration status, and differences in cultural norms from the dominant U.S. culture. Participants also recommended adding content on interactions with police, gender dysphoria, rejection by family and friends, and trauma-informed practices they deemed important to their lived experiences as transgender Latinas.

Relatedly, participants described wanting consistent ways to connect with other transgender Latinas as the lack of social support and culturally relevant information was noted as a barrier to accessing services. Hence, a social media component was recommended, whereby intervention participants would be connected throughout the program via a WhatsApp Group to enhance connectivity among participants and sustain support throughout the program.

The resulting adapted intervention directly addresses the lack of social support and connection within the community. Furthermore, it equips participants with knowledge about their rights and skills to navigate discrimination and stigma associated with their identities. These tailored aspects of the intervention make it well-suited to address the challenges faced by transgender Latina women in the context of HIV prevention and care.

In response to participant feedback during the various steps of adaptation, the program was titled *Somos Chingonas*, translating to “We are badass” or “We are strong and capable women.” The term *chingona* was chosen for its colloquial nature, universal understanding across diverse Latina origins, and its representation of a confident and assertive woman. This phrase encapsulates the intervention’s goal of promoting empowerment and self-assuredness in the context of HIV prevention, as well as emphasizing its community-oriented nature and specific adaptation for transgender Latina women.

### Topical expert adaptations

Feedback from topic experts (*n* = 5) additionally emphasized the importance of a status-neutral approach to decrease HIV stigma. Given the high risk of HIV transmission in this population, experts recommended shifting the focus from the original EBI’s outcome of condomless sex to outcomes that also included antiretroviral medication use (i.e., condomless sex without concurrent pre-exposure prophylaxis or antiretroviral therapy use).

**FIG. 1.**
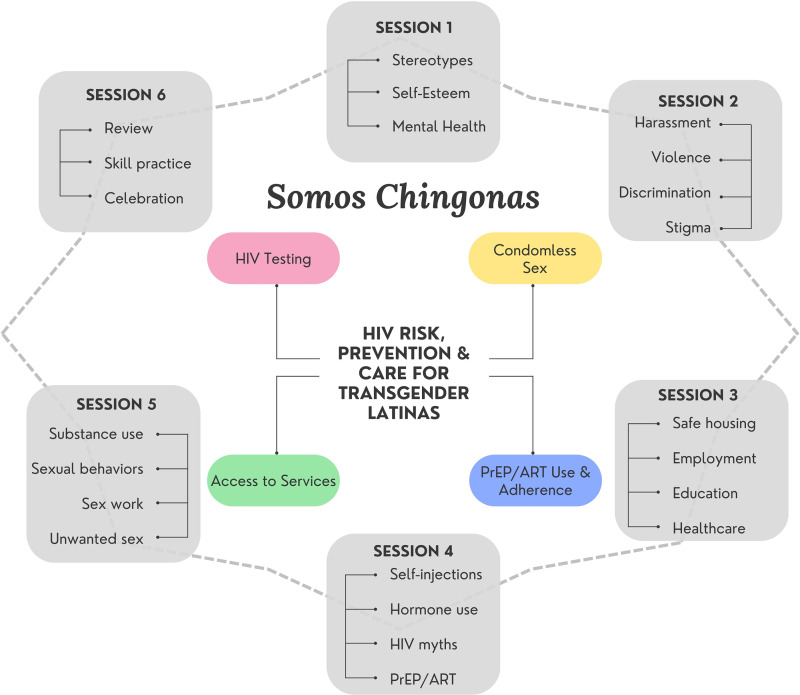
Key topics and session components of adapted intervention.

Table 2 outlines the EBI adaptations and changes integrated after theater testing and expert feedback. The aim of this step was to create a thoroughly adapted intervention tailored for transgender Latina women in preparation for pilot testing. Figure 1 illustrates the key topics and session components of the adapted *Somos Chingonas* intervention. In our study, findings from each of the adaptation steps generally converged. In cases of conflicting perspectives, the study team deferred to the expertise and perspectives of community members, given their lived experiences. The overarching objective of the adapted intervention is to positively impact HIV testing and service engagement, increase use and adherence to pre-exposure prophylaxis and antiretroviral therapy, and reduce condomless sex among transgender Latina women.

## Discussion

To address the lack of adequate HIV prevention interventions specifically designed for transgender Latina women in the United States,^6^ our study employed the ADAPT-ITT method^9^ to adapt an existing EBI for this demographic. We ensured accessibility by adapting all materials and activities in both English and Spanish, and this article provides an overview of the adaptation and tailoring process.

Our study underscored the critical role of community involvement and perspectives in developing interventions tailored to specific racial, ethnic, and gender minority communities. A thorough needs assessment with transgender Latina women provided valuable insights into the unique behavioral and psychosocial risks that increase vulnerability to HIV among this population, as well as their preferences regarding the cultural adaptation of intervention content and delivery methods.

The incorporation of feedback from both the target population and topical experts within the transgender Latina community played a pivotal role in the last step of our adaptation process integration. Community leaders and organization staff whom we interviewed validated the appropriateness of our adaptations and their resonance with the cultural nuances and perspectives of the community. Particularly significant was the integration of content like trauma-informed practices, addressing the unique experiences of the transgender Latina community. These aspects were among the reasons cited by topical experts for why they viewed the adapted intervention as a good fit.

### Limitations

The adapted intervention did not include the perspectives and experiences of all transgender Latina women, given the study’s sample and overall approach. Despite efforts to include diverse participants, there may have been limitations in representing the full diversity of transgender Latina women, including variations in socioeconomic status and cultural backgrounds. Furthermore, adaptations focused on foreign-born transgender Latinas, given their higher risk for poor health due to challenges related to immigration status, which may have limited the adaptations’ relevance for U.S.-born transgender Latinas. While efforts were made to ensure linguistic adaptation, language barriers may still exist for individuals who identify as Latina but do not speak Spanish, potentially limiting the intervention’s reach.

## Conclusion

Our study contributes to the literature on intervention adaptation by providing a detailed account of the ADAPT-ITT process,^9^ as applied to an evidence-based HIV prevention intervention for transgender Latina women. The integration of community voices resulted in a tailored intervention to address the specific needs of this population, offering a promising avenue for more effective and culturally competent HIV prevention and care strategies. Future research will focus on the training and testing phases of the ADAPT-ITT process to evaluate the impact of the adapted intervention on HIV prevention and care outcomes within the transgender Latina community.

## Authors’ Contributions

All authors contributed to the conceptualization and conducting of the study and analysis of findings. J.J.L. led the interpretation and writing of this article with support from J.L.V. and S.M.G. All authors reviewed and edited the article.

## Abbreviations

ADAPT-ITT: assessment, decision, adaptation, production, topical experts, integration, training, testing
ART: antiretroviral therapy
CDC: Centers for Disease Control and Prevention
EBI: evidence-based intervention
HIV: human immunodeficiency virus
LGBTQ+: lesbian, gay, bisexual, transgender, and queer
M: mean
PrEP: pre-exposure prophylaxis
